# Therapeutic effects of *Medicago sativa* against cyclophosphamide-induced toxicity in rats

**DOI:** 10.22038/AJP.2023.22911

**Published:** 2024

**Authors:** Vajihe Rouki, Mohammad Hossein Boskabady, Narges Marefati, Reyhaneh Sotoudeh, Zahra Gholamnezhad

**Affiliations:** 1 *Department of Physiology, Faculty of Medicine, Mashhad University of Medical Sciences, Mashhad, Iran *; 2 *Applied Biomedical Research Center, Mashhad University of Medical Sciences, Mashhad, Iran*; 3 *Department of Physiology and Medical Physics, Faculty of Medicine, Baqiyatallah University of Medical Sciences, Tehran, Iran*

**Keywords:** Cyclophosphamide, Medicago sativa, Myelosuppression Thrombocytopenia, Oxidative stress

## Abstract

**Objective:**

*Medicago sativa* (*M. sativa*) has been traditionally used for treating anemia; therefore, *M. sativa* hydro-ethanolic extract therapeutic effects against cyclophosphamide (CP) -induced hematologic and liver toxicity were examined.

**Materials and Methods:**

Thirty male Wistar rats were randomly divided to control (saline); CP (100 mg/kg, day 1-3, subcutaneously); CP+ *M. sativa* 200 mg/kg (MS 200); CP+ *M. sativa* 400 mg/kg (MS 400); CP+ dexamethasone (0.1 mg/kg), (all groups n=6). Treated animals received *M. sativa* or dexamethasone by gavage from days 7-14. On days 0, 7, and 14, hematologic parameters, and on the 14th day, serum and liver tissue oxidative stress markers including nitric oxide, malondialdehyde (MDA) and total thiol levels, superoxide dismutase (SOD) and catalase (CAT) activities, serum lipids, and liver enzymes were measured.

**Results:**

Animal weight, platelet, white blood cells, and red blood cells counts, hemoglobin and hematocrit as well as thiol, SOD, and CAT activities in serum and liver tissue were significantly reduced, but serum nitric oxide, MDA, total cholesterol, triglycerides, low-density lipoproteins levels, and liver enzymes were increased in the CP group compared to the control group (p<0.05 to p<0.001). Administering *M. sativa* extract (400 mg/kg) significantly enhanced platelet count, and SOD and CAT activities and inhibited all of the CP toxic effects, while dexamethasone improved platelet count and oxidative stress markers compared to the CP group (p<0.05 to p<0.001).

**Conclusion:**

The extract of *M. sativa* (400 mg/kg) showed therapeutic effects against the CP-induced myelosuppression and thrombocytopenia and improved oxidative stress markers which were comparable to the effect of dexamethasone.

## Introduction

Chemotherapy has been used to treat various cancers for many years. Cyclophosphamide (CP) is a chemotherapeutic alkylating agent in the family of anti-neoplastic drugs. CP is well-known for its anticancer effect, immunosuppressive activity (in organ transplantation) and treatment of autoimmune diseases like systemic lupus erythematosus, rheumatoid arthritis, and multiple sclerosis (Nie et al., 2010; Cengiz et al., 2020). Despite the pharmacological efficacy of CP, it causes serious side effects including bone marrow suppression (myelosuppression), cardiac, pulmonary, renal and hepatic toxicity, hair loss, bladder bleeding, and immunosuppression (Fraiser et al., 1991; Cengiz et al., 2019). Its toxic metabolites acrolein and phosphoramide mustard cause irreversible cross-linkages in the DNA strands that lead to cell death not only in tumors but also in normal tissue; this has limited its use (Fraiser et al., 1991). Therefore, this compound is used to establish an effective model for the study of the pharmacodynamics of drugs and plant compounds in the treatment of chemotherapy toxicity (Nie et al., 2010; Khazaei et al., 2020).

*Medicago sativa* (*M. sativa*) with a common name of alfalfa is a plant belonging to the Fabaceae family. *M. sativa* is a medicinal plant and functional food with several pharmacological effects. Due to medicinal properties and nutrients, this plant has been considered a dietary supplement and herbal medicine (Mikaili and Shayegh, 2011; Karimi et al., 2013). *M. sativa* is rich in vitamins A, C, E, and K (Stochmal et al., 2001) and contains pharmacologically active substances such as alkaloids, flavonoids, steroids, and minerals. In traditional medicine, this plant has a warm nature and has been used to treat coagulation disorders, anemia, and arthritis (Basch et al., 2003). Experimental studies have demonstrated the effect of *M. sativa* on tissue repair, and acceleration of joint cartilage recovery in laboratory animals. The plant also might decrease hemoglobin glycolysis, suppress platelet aggregation and exert hypolipidemic, anti-inflammatory, and antioxidant properties (Basch et al., 2003; Hong et al., 2009; Rafiei and Khayatzadeh, 2012; Karimi et al., 2013; Hosseini et al., 2015; Shirani et al., 2015). The anticoagulant effects of this plant have been explained by several mechanisms such as high content of vitamin K, and inhibition of adenosine diphosphate-induced platelet aggregation, and thromboxane synthesis in platelets (Pierre et al., 2005). These pharmacological effects and medicinal properties of *M. sativa* had been shown in studies using different types of plant leaves or seeds extracts including aqueous, alcoholic, acetate, ether, butanol, and others. Hydro-ethanolic extract of the plant might contain both water (i.e. vitamin C) and lipid (i.e. phytoestrogen) soluble compounds of the *M. sativa* leaves (Al-Snafi et al., 2021). 

However, to the best of our knowledge, there is no finding about the therapeutic effects of *M. sativa *on CP toxicity. Thus, in this study, the therapeutic effects of *M. sativa* hydro-ethanolic extract on bone marrow suppression, liver toxicity, and oxidative stress induced by CP were investigated.

## Materials and Methods

**Preparation of** ***M. sativa*** **extract**

The plant was freshly harvested from a farm in Gonabad, south east of Khorasan Razavi province, Iran and was identified (Specimen number: 38075) by a botanist in the herbarium center of Ferdowsi University (Mashhad, Iran).

For hydro-ethanolic extract preparation, 200 g of chopped *M. sativa* aerial parts was macerated in 800 ml of ethanol/water (50:50% v/v) for 72 hr at 40°C. Then, the solvent was totally removed by rotary evaporation under reduced pressure. The extraction yield was 23% and the extract was stored at 4°C in a dark closed container. 

**Materials and drugs**

CP was purchased from Baxter Company (Germany), dexamethasone (Dexa) was obtained from Sigma (St. Louis, MO, Germany), and for biochemical assessments, agents were purchased from Merck Company. Pars Azmoon Company (Iran, Tehran) kits were used for determination of liver enzymes (alanine transaminase (ALT), aspartate aminotransferase (AST), and alkaline phosphatase (ALP)), total cholesterol (TC), triglycerides (TG), low-density lipoproteins (LDL-C), and high-density lipoprotein (HDL-C).

**Animals**

Thirty adult male Wistar rats, weighing 200-250 g, were obtained from animal house of Mashhad University of Medical Sciences, Mashhad, Iran. Animals were housed under standard conditions (12 hr light/dark cycle and temperature 22-24°C) and their food and water were available *ad libitum* during the experiment. All procedures done in this search followed national guidelines for animal studies and the study protocol was approved by the Ethics Committee for Animal Research of Mashhad University of Medical Sciences, Mashhad, Iran (IR.MUMS.fm.REC.1395.13).

**Experimental procedure**

The study was conducted in a period of 14 days, and animals were randomly divided into 5 groups (n=6) as follows (Khazaei et al., 2020): 

Control (saline); CP (100 mg/kg subcutaneous **(**SC) from day 1 to 3) (Kristiana et al., 2013); MS 200 and MS 400 groups that received CP and *M. sativa* 200, 400 mg/kg/day respectively (Gholamnezhad et al., 2023) and Dexa group which received CP and dexamethasone (0.1 mg/kg). Treated groups received the extract or dexamethasone via gavage from day 7 to 14. On days 0 and 7 of the study, animals were anesthetized using diethyl ether and blood samples were collected from the retro-orbital sinus for measurement of hematologic parameters. At the end of the study (i.e. day 14), animals were euthanized under deep anesthesia (ketamine 80 mg/kg + xylazine 8 mg/kg, intraperitoneally) and blood samples were taken from the heart for measurement of hematologic parameters; serum was separated for evaluation of lipid profile, and liver enzymes and oxidative stress markers levels including malondialdehyde (MDA), total thiol, superoxide dismutase (SOD) activity, catalase (CAT) activity and NO metabolite (NO_2_^-^). The liver was also isolated for determination of hepatic levels of MDA, SOD, CAT, and total thiol.

**Biochemical assessments**

**MDA**

MDA as an indicator of lipid peroxidation, was measured based on the MDA reaction with thiobarbituric acid (TBA), producing a pink complex with a peak absorbance at 535 nm (Kaveh et al., 2017).

**Measurement of total thiol content**

Total thiol content was measured by the method of Ellman. SH groups produce a yellow complex which has a peak absorbance at 412 nm (Ellman, 1959).

**SOD activity**

SOD activity was measured based on a procedure described by Madesh and Balasubramanian. The procedure involves production of superoxide through auto-oxidation of pyrogallol. In the presence of SOD, this process is inhibited, therefore SOD activity is indirectly assayed at 570 nm (Madesh and Balasubramanian, 1998).

**CAT activity**

The activity of CAT was measured according to the method of Aebi, based on the rate of decomposition of hydrogen peroxide (H_2_O_2_) by CAT as determined using a spectrophotometer at 240 nm (Aebi, 1984).

**NO metabolite**

NO metabolite (nitrite) was measured based on the reaction between Griess reagent, sulfanilamide and naphthylethylenediamine (NED) solutions at 520 nm (Hosseini et al., 2014).

**Statistical analysis**

Results are presented as mean ± standard error of the mean (SEM) and were analyzed using Instat software. If the differences among the SDs were not significant, data were compared using one-way ANOVA followed by a Tukey's *post hoc* comparisons test, otherwise Kruskal-Wallis test was used. The impact of time on each group of data was evaluated by repeated measurement of ANOVA. Statistical significance was considered at p<0.05.

## Results

**Animals’ weight gain changes**

**Figure 1:**
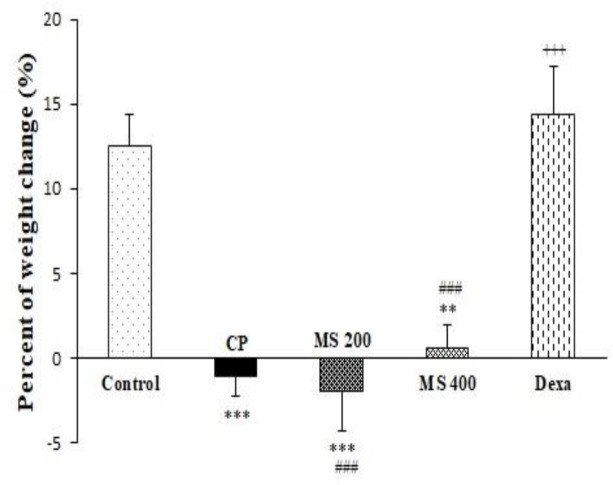
Weight changes (%) of the control, cyclophosphamide (CP), *M. sativa* extract (MS at doses of 200, and 400 mg/kg) and dexamethasone (Dexa) treated groups (n= 6 in each). Data are presented as mean±SEM. Comparisons were made using one-way ANOVA followed by a Tukey's *post hoc*. **p<0.01 and ***p<0.001; significant differences compared to the control group; ^+++^p<0.001: significant difference compared to the CP group; and ^###^p<0.001: significant differences compared to the Dexa group.

Three days of CP injection reduced animals' weight gain. Percent of weight gain in CP and MS 200 and 400 mg/kg treated groups was significantly lower, but in Dexa group was higher than the control group (p<0.01-p<0.001). Weight gain increased significantly in dexamethasone-treated group compared to the CP group (p<0.001); however, weight gain changes in MS 200 and 400 groups were lower than dexamethasone-treated group (p<0.001 for both groups, Figure 1). 

**Hematological parameters** **changes**

There were no significant differences in platelet count among days 0, 7, and 14 in the control group. Platelet count of the CP group on days 7 and 14, was significantly lower than that of day 0 of the CP group and similar days in the control group (p<0.001 for all). Within group comparison showed that in all treated groups, on day 7, platelet count was lower than that of day 0 (p<0.001 for all).

**Figure 2:**
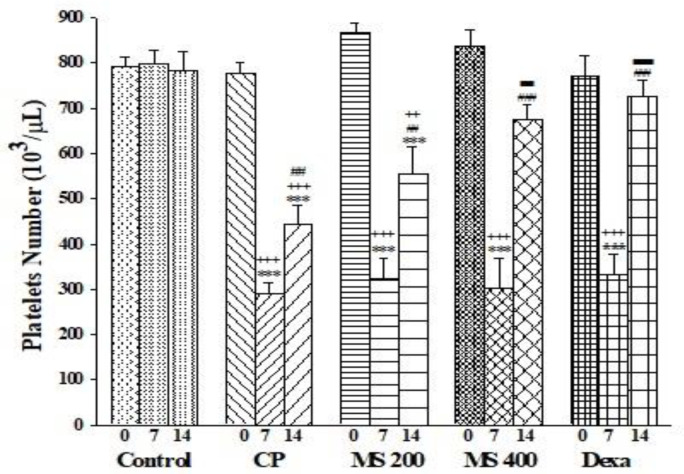
Platelets number on days 0, 7, and 14 in the control, cyclophosphamide (CP), *M. sativa* extract (MS, at doses of 200, and 400 mg/kg) and dexamethasone (Dexa) treated groups (n= 6 in each). Data are presented as mean±SEM. Within group comparison (between day 0, 7 and 14) was made by repeated measurement of ANOVA, and between groups comparisons was made using one-way ANOVA followed by a Tukey's *post hoc*. ***p<0.001 shows significant differences as compared to day 0; ^++^p<0.01 and^ +++^p<0.001 show significant differences as compared to the control group; ^##^p<0.01 and ^###^p<0.001 show significant differences as compared to day 7; ^■■^p<0.01, ^■■■^p<0.001 show significant differences as compared to the CP group.

Moreover, on day 7, the platelet count of all treated groups was lower than that of the control group (p<0.001 for all). In the MS 200 group, the platelet count on day 14 was lower that of day 0 and 14 of the control group (p<0.01 for both). Platelet number on day 14 in all treated groups was significantly higher than that of day 7 (p<0.05 to p<0.001). The platelet count on day 14 in the MS 400 and Dexa groups was significantly higher than the CP group (p<0.01 for both, Figure 2).

The WBC count in the CP group and all treated groups was decreased significantly on day 7 and 14 compared to day 0 of the experiment (p<0.05 to p<0.001). In the CP and MS 400 groups, the WBC count on day 14 was higher than day 7. Between groups comparison showed that the WBC count of the CP and all treated groups on day 7 and 14 was significantly lower than the similar day of the control group (p<0.01 to p<0.001, Table 1). 

The RBC count on day 7 and 14 was significantly lower compared to day 0 of the experiment, in the CP and all treated groups (p<0.05 to p<0.001). The RBC counts in the CP and all treated groups, on the day 7 and in the CP, MS 200 and MS 400 groups on day 14 were significantly lower than that of the same day in the control group (p<0.05 to p<0.01, Table 1).

Within group comparison showed that on day 7 and 14, the Hb concentration was significantly lower than that of day 0 in the CP, and all treated groups (p<0.05 to p<0.001). In addition, the Hb concentration of MS 400 group on day 14 was higher than day 7. Between groups comparison showed that the Hb concentration on day 7 in the CP and all treated groups and on day 14 in the CP and MS 200 groups was significantly lower than that of the same day in the control group (p<0.05 to p<0.001, Table 1).

On day 7 and 14, the Hct (%) was decreased significantly in the CP, MS 200, MS 400 and Dexa groups compared to day 0 (p<0.05 to p<0.001). Moreover, in the CP, MS 400 and Dexa groups, Hct level on day 14 was significantly higher than that of day 7 (p<0.05 for all groups). Between groups comparison showed that the Hct level on day 7 in the CP and all treated groups and on day 14 in the CP and MS 200 groups was significantly lower than that of the same day in the control group (p<0.01 to p<0.001, Table 1).

The MCV indices on day 7 and 14 in the MS 200 group were higher than that of day 0, and on day 14 in the MS 400 group were higher than that of day 0 and 7 of the same group (p<0.05 to p<0.001). There were no significant differences between groups or within groups with regard to other corpuscular indices (Table 1). 

**S** **erum** **concentrations of liver enzymes and lipids**

At the end of the study, in the CP group, HDL level was decreased and serum concentration of liver enzymes (AST and ALT), triglycerides, cholesterol, and LDL were increased significantly in comparison with the control group (p<0.05 to p<0.001). The cholesterol and triglycerides levels in Dexa group were significantly higher than the control, CP, MS 200 and 400 groups (p<0.01 to p<0.001). While in the MS 200 and 400 groups, cholesterol level was lower than that of the CP group (p<0.01 for both), in the MS 200, it was higher than the control group (p<0.05). The HDL levels in the Dexa, MS 200 and MS 400 groups were lower but the LDL level in the Dexa group was higher than the control group (p<0.05 to p<0.001). The serum concentration of ALP in the Dexa group was significantly higher than that of the control, CP, MS 200 and 400 groups (p<0.01 to p<0.001). Although there were no significant differences in serum AST concentration among the control, MS 200 and MS 400 groups, ALT concentration was increased in the MS 200 and MS 400 groups compared to the control group (p<0.05). In the Dexa group, AST level was higher than the control group (p<0.01). In addition, MS treatment decreased AST compared to the CP group (p<0.05 to p<0.01, Table 2).

**Serum and liver MDA concentrations, thiol content, and SOD and CAT activities**

**Figure 3:**
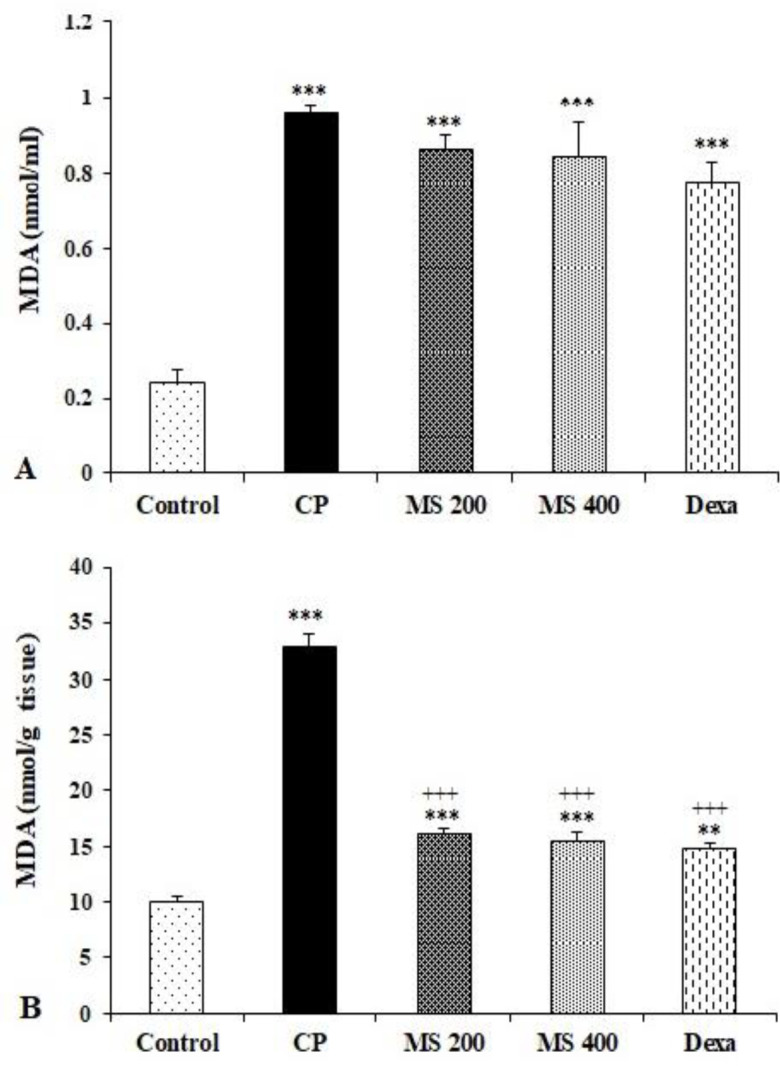
Serum (A) and liver (B) MDA levels in the control, cyclophosphamide (CP) and *M. sativa* extract (MS, at doses of 200, and 400 mg/kg) and dexamethasone (Dexa) (n= 6 in each). Data are presented as mean±SEM. Comparisons were made using one-way ANOVA followed by a Tukey's *post hoc*. ***p<0.001 shows significant differences as compared to the control group and ^+++^p<0.001 shows significant differences as compared to the CP group.

Serum level of MDA as an end product of lipid peroxidation, was increased significantly in the CP, *M. sativa* and dexamethasone treated groups (p<0.001 for all) compared to the control group. There was no significant difference in the serum level of MDA between *M. sativa* and dexamethasone treated groups (Figure 3A). 

**Table 1:** Levels of hematologic parameters in different groups on days 0, 7, and 14 of the experiment

MCHC (g/dl)	MCH (pg)	MCV (fl)	Hct (%)	Hb (g/dl)	RBC (10^6^/l)	WBC (10^3^/l)	Groups Variables
33.09±0.44	22.56±0.81	54.53±1.49	42.7± 0.94	14.13±0.34	7.83±0.25	18.86±0.46	Control- day 0
33.77±0.66	18.20±0.66	53.88±1.56	40.96±1.06	13.83±0.32	7.60±0.24	18.08±0.66	Control-day 7
32.11±0.82	17.78±0.50	55.38±1.60	41.97±0.78	13.48±0.25	7.58±0.31	16.30 ±0.95	Control-day 14
32.75± 0.74	18.10±0.56	55.30±1.35	42.52± 0.88	13.92±0.25	7.69±0.24	17.50±1.55	CP-day 0
31.25±0.76	20.80±0.79	57.93±1.59	29.03±1.04^***+++^	10.45±0.28^***+++^	5.01±0.57^***++^	3.16±0.88^***+++^	CP-day 7
32.37± 0.48	18.18±0.58	56.21±0.87	33.43±0.99^***#+++^	10.82±0.47^***++^	5.78 0.23^*+^	9.360.86^***##+++^	CP-day 14
33.92±0.49	17.88±0.68	52.40±0.44	43.92±1.14	14.90±0.21	8.33±0.2	18.800.68	MS 200-day 0
30.22±0.75	18.78±0.48	62.21±1.02^***++^	32.10±1.52^***+++^	9.70±0.72^***+++^	5.16±0.37^***++^	3.80±0.53^***+++^	MS 200-day 7
29.90±0.83	17.97±0.56	60.05±1.10^***^	35.43±1.73^**++^	10.60±0.85^***++^	5.90±0.55^**+^	6.25±0.78^***+++^	MS 200-day 14
31.98±0.89	19.18±0.62	59.97±1.21	44.38±1.37	14.20±0.30	7.40±0.22	18.430.66	MS 400-day 0
29.87±0.81	17.02±0.58	56.97±1.31	32.25±1.46^***+++^	9.63±0.45^***+++^	5.66±0.41^*+^	3.38±0.65^***+++^	MS 400-day 7
30.68±0.88	19.78±0.58	64.50±0.95^*##^	38.12±0.87^**#^	11.70±0.27^***##^	5.91±0.46^*+^	9.65±1.18^*#++^	MS 400-day 14
33.83±0.82	19.45±0.55	57.45±0.95	43.73±1.35	14.80± 0.56	7.61±0.29	18.600.53	Dexa-day 0
33.10±0.82	18.22±0.75	55.10±1.24	31.08±1.28^***+++^	10.27±0.32^***+++^	5.64±0.38^**+^	5.48±0.75^***+++^	Dexa-day 7
32.25±0.76	18.73±0.73	58.08±1.19	36.83±1.6^**#^	11.88±0.49^**^	6.34±0.26^*^	9.18±1.35^**+++^	Dexa-day 14

Hematological parameters on days 0, 7, and 14 in cyclophosphamide (CP), *M. sativa* extract (MS, at doses of 200, and 400 mg/kg) and dexamethasone (Dexa) groups (n=6 in each). Data are presented as mean±SEM. Within group comparison (between day 0, 7 and 14) was made by repeated measurement of ANOVA, and between groups comparisons was made using one-way ANOVA followed by a Tukey's *post hoc*. *p<0.05, **p<0.01 and ***p<0.001 show significant differences as compared to day 0; ^##^p<0.01 and ^###^p<0.001 show significant differences as compared to day 7;^ ++^p<0.01 and^ +++^p<0.001 show significant differences as compared to the control group.

**Table 2:** Liver enzymes and serum lipid levels in different groups

Parameter/ Groups	Cholesterol (mg/dl)	Triglycerides (mg/dl)	HDL (mg/dl)	LDL (mg/dl)	ALP (U/L)	AST (U/L)	ALT (U/L)
Control	49.17±2.43	54.83±5.32	34.33±2.86	10.6±1.07	296.33±40.78	45.53±7.54	28.50±6.50
CP	95.50±5.71^***^	97.30±9.12^*^	14.47±3.18^***^	22.23±2.59^*^	672.00±71.47	149.50±17.62^***^	72.30±11.30^**^
MS 200	67.83±5.48^*+++^^###^	88.17±7.53^###^	16.10±2.34^***^	16.23± 1.75	564.67± 112.64^###^	76.50± 9.55^++^	63.67±6.32^*^
MS 400	62.50± 2.53^+++^^###^	81.17±6.75^###^	21.50±2.32^*^	14.20± 2.29^#^	584.67±77.20^###^	94.00± 10.88^+^	58.50± 7.08^*^
Dexa	128.83±3.94^***+++^	235.17±13.81^***+++^	18.17±2.36^**^	25.00± 3.74^**^	1298 ±143.59^***+++^	113.17± 15.54^**^	51.67± 4.26

Data are presented as mean±SEM. Comparisons were made using one-way ANOVA followed by a Tukey's *post hoc*. Cyclophosphamide (CP) and *M. sativa* extract (MS, at doses of 200, and 400 mg/kg) and dexamethasone (Dexa) (n= 6 in each). *p<0.05 and **p<0.01 show significant differences as compared to the control group; ^+^p<0.05 and ^++^p<0.01 show significant differences as compared to the CP group. ###p<0.001 shows significant differences compared to the Dexa group.

Liver MDA concentration was significantly increased in the CP, *M. sativa* and dexamethasone treated groups (p<0.01 to p<0.001) compared to the control group. However, treatment of animals with *M. sativa* and dexamethasone significantly decreased the MDA concentration in comparison to the CP group (p<0.001, Figure 3B).

**Figure 4:**
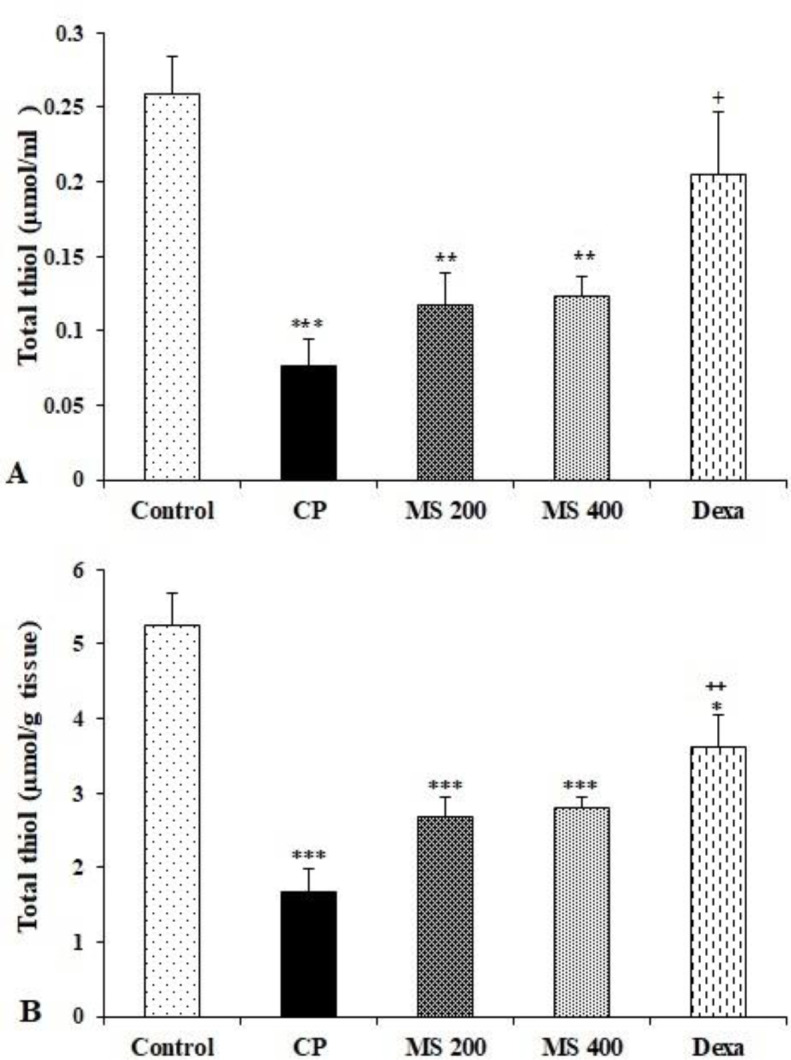
Serum (A) and liver (B) total thiol levels in the control, cyclophosphamide (CP), and *M. sativa* extract (MS, at doses of 200, and 400 mg/kg) and dexamethasone (Dexa) (n= 6 in each). Data are presented as mean±SEM. Comparisons were made using one-way ANOVA followed by a Tukey's *post hoc*. * p<0.05, **p<0.01 and ***p<0.001 show significant differences as compared to the control group; ^+^p<0.05 and^ ++^p<0.01 show significant differences as compared to the CP group.

**Figure 5:**
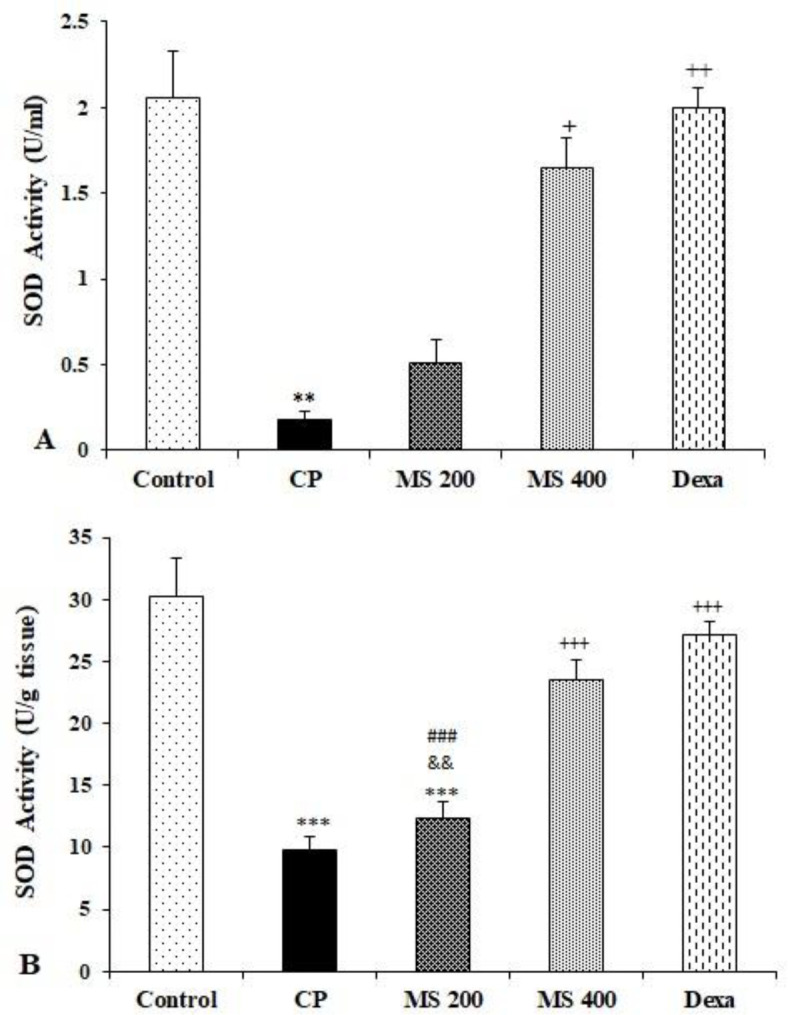
Serum (A) and liver (B) SOD activity in the control, cyclophosphamide (CP), and *M. sativa* extract (MS, at doses of 200, and 400 mg/kg) and dexamethasone (Dexa) (n= 6 in each). Data are presented as mean±SEM. Comparisons were made using Kruskal Wallis with *post-hoc Dunn's* test (A) or one-way ANOVA followed by a Tukey's *post hoc* (B). ^**^p<0.01 and ^***^p<0.001 show significant differences as compared to the control group; ^+^p<0.05, ^++^p<0.01 and ^+++^p <0.001 show significant differences as compared to the CP group; ^###^p<0.001 shows significant differences as compared to the Dexa group; ^&&^p<0.01 shows significant differences as compared to the MS 400 group.

The result showed a significant reduction in serum total thiol content in the CP and *M. sativa* treated groups compared to the control group (p<0.001 for CP, and p<0.01 for others). Serum total thiol content in the Dexa group was significantly higher than the CP group (p<0.05, Figure 4A). The liver total thiol content in the CP, *M. sativa* and dexamethasone treated groups was significantly lower than the control group (p<0.05 for Dexa and p<0.001 for others). Moreover, liver total thiol content in the Dexa group was significantly higher than the CP group (p<0.01, Figure 4B). There was a marked reduction in serum SOD activity in the CP group compared to the control group (p<0.01). Although *M. sativa* administration partially attenuated the effect of CP, serum SOD activity in *M. sativa* treated groups remained significantly lower than the control group but in the MS 400 and Dexa groups, it was higher than the CP group (p<0.05 to p<0.01, Figure 5A). 

Liver SOD activity in the CP and MS 200 groups was significantly lower than the control group (p<0.001); however, in the MS 400 and Dexa groups, it was significantly higher than the CP group (p<0.001 for both). The SOD activity in the MS 200 group was lower than both MS 400 and Dexa groups (p<0.01 to p<0.001, Figure 5B). 

**Figure 6:**
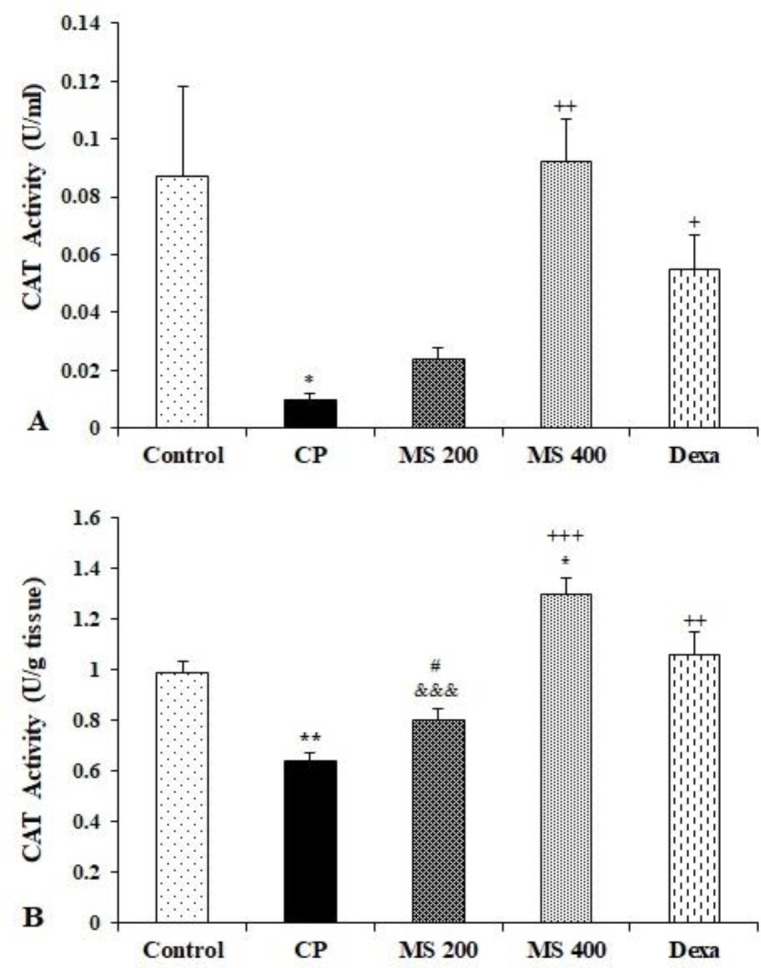
Serum (A) and liver (B) catalase (CAT) activity in the control, cyclophosphamide (CP), and *M. sativa* extract (MS, at doses of 200, and 400 mg/kg) and dexamethasone (Dexa) (n= 6 in each). Data are presented as mean±SEM. Comparisons were made using using Kruskal Wallis with *post-hoc Dunn's* test (A) or one-way ANOVA followed by a Tukey's *post hoc* (B). *p<0.05 and **p<0.01 show significant differences as compared to the control group;^ +^p<0.05, ^++^p<0.01 and ^+++^p<0.001 show significant differences as compared to the CP group; and ^#^p<0.05 shows significant differences as compared to the Dexa group.^ &&&^p<0.001 shows significant differences as compared to the MS 400 group.

The serum catalase (CAT) activity in the CP group was significantly lower compared to the control group (p<0.05). Treatment of animals with dexamethasone and *M. sativa* (400 mg/kg) increased the CAT activity compared to the CP group (p<0.05 to p<0.01, Figure 6A). 

The liver tissue CAT activity was significantly lower in the CP group and higher in MS 400 group compared to the control group (p<0.05 to p<0.01). Treatment of animals with MS 400 and dexamethasone significantly increased the CAT activity compared CP groups (p<0.01 to p<0.001). However, there were no significant differences in the CAT activity between CP, MS 200, and Dexa groups and the control group. The CAT activity of the MS 200 group was significantly lower than both MS 400 and Dexa groups (p<0.001 and p<0.05 respectively, Figure 6B). 

**Serum level of NO metabolite**

**Figure 7:**
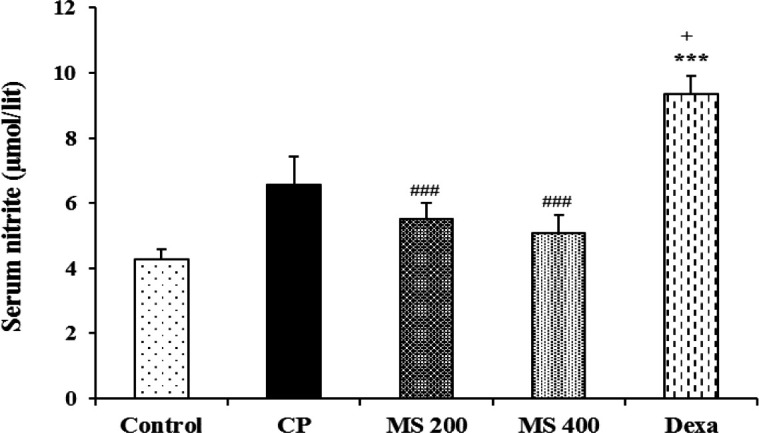
Serum nitrite level in the control, cyclophosphamide (CP), and *M. sativa* extract (MS, at doses of 200, and 400 mg/kg) and dexamethasone (Dexa) (n= 6 in each). Data are presented as mean±SEM. Comparisons were made using one-way ANOVA followed by a Tukey's *post hoc*. ***p<0.001 shows significant differences as compared to the control group; ^+^p<0.05 shows significant differences as compared to the CP group; and ^###^p<0.001 shows significant differences as compared to the Dexa group.

The serum NO metabolites in the Dexa group increased significantly compared to the control and CP groups (p<0.001 and p<0.05, respectively). The NO metabolites level in the MS 200 and MS 400 groups was lower than the Dexa group (p<0.001 for both, Figure 7).

## Discussion

The results of the present study confirm the previous findings that CP injection causes weight loss, thrombocytopenia, leukopenia, anemia, hyperlipidemia, and liver toxicity and increases the levels of oxidative stress markers (Akhter et al., 2015; Gholamnezhad et al., 2023; Parandin et al., 2023). In another study, administration of CP at a dose similar to that used in the present study, caused leukopenia and decreased mean blood cell volume (Shirani et al., 2015). Moreover, CP injection (15 mg/kg, i.p.) for fifteen consecutive days significantly decreased the number of blood platelet, WBC, RBC, and the levels of Hct, Hb, MCH, MCV, and MCHC (Mirazi et al., 2021). In the present study, although CP induced hematopoietic tissue suppression, the level of MCH, MCV, and MCHC did not significantly change, indicating induction of normochromic normocytic anemia in the CP group animals.

In patients receiving CP injection similar symptoms including weight loss, anorexia, alopecia, thrombocytopenia, anemia, and leukopenia are reported (Fraiser et al., 1991). Different mechanism such as apoptosis, oxidative stress, and inflammation have been proposed for toxic effects of CP especially on bone marrow suppression (Abdelzaher et al., 2020). In this study, elevation of MDA and NO metabolite levels, as well as reduction of CAT and SOD activity, and content of total thiol in serum and liver tissue, indicates the excessive formation of reactive oxygen species (ROS) by CP alkylating metabolite. Oxidative stress not only affects cancer cells in patients receiving chemotherapy, but also damages cellular DNA, lipids and proteins in normal tissue and bone marrow hematopoietic cells (Ahlmann and Hempel, 2016). It seems that CP could influence both production and destruction of normal blood cells through apoptosis and oxidative stress, which resulted in thrombocytopenia, leukopenia, and anemia (Basu et al., 2015; Cengiz et al., 2020). Therefore, antioxidant-rich compound might be able to reduce the toxic effect of CP metabolite during chemotherapy (Abdelfattah-Hassan et al., 2019).

*M. sativa* as a valuable herbal plant is rich in amino acids (lysine, arginine, histidine, adenine, phenylalanine, asparagine, and cysteine), phytoestrogen (saponins), phenolic and flavonoid compounds, vitamins (A, C, E, and K), enzymes (amylase, pectinase, and invertase) and other nutrients (magnesium and iron) (Bresson, 2009). It had been shown that the plant estrogens inhibit endoplasmic reticulum stress-induced apoptosis and they are effective against malignancies like acute myeloid leukemia and breast cancer due to anti-proliferative properties (Yang et al., 2019). Therefore, to evaluate the therapeutic effects of *M. sativa* against CP toxicity, the animals were treated with the plant extract (200 and 400 mg/kg) during days 7 to 14 of the experiment and the results were compared with dexamethasone as an adjuvant drug for CP. 

*M. sativa* administration did not restore CP-induced animal weight loss, while dexamethasone increased animal’s weight in line with previous findings (Wong et al., 2018).

One-week administration of the *M. sativa* extract ameliorated the CP-induced bone marrow suppression and the related leukopenia, thrombocytopenia and normocytic anemia. Treatment of animal with MS 400 significantly improved the Hb and Hct levels, and MCV indices compared to day 7, but the RBC count was not changed significantly. These findings might show the higher impact of the plant against CP toxicity on RBC maturation than RBC progenitor proliferation. It is believed that the presence of antioxidants including polyphenols, saponins, and alkaloids, as well as nutrients like polysaccharides, and the amino acids in the herbal plant might suppress the toxic effect of CP on bone marrow and restore its ability to produce platelets and other blood cells (Patil et al., 2013). In addition,* M. sativa* affects the coagulation system via its high content of vitamin K, inhibition of ADP and collagen induced platelet aggregation, and inhibition of thromboxane synthesis in platelets (Pierre et al., 2005). The plant extracts administration has been reported to induce leucocytosis and neutrophilia probably due to their ability to activate macrophages, and increase the secretion of substances such as colony activating factor and interleukin-1 (Kaneko et al., 1999; Haque and Ansari, 2014).

Several studies reported other side effects of CP including cardiac, liver and metabolic, renal, and gastrointestinal toxicities, infertility and gonadal disorders (Ghosh et al., 1999; Mythili et al., 2006; Zarei and Shivanandappa, 2013). In the present study, administration of CP increased the serum levels of liver enzymes AST, ALT, and ALP, and induced oxidative stress markers indicating liver damage caused by toxic metabolites of this drug. CP even at low doses induces hepatotoxicity which leads to hepatocellular damage and elevation of serum levels of cytosolic enzymes (AST, ALT, ALP, and LDH) (Senthilkumar et al., 2006; Subramaniam et al., 2013; Zarei and Shivanandappa, 2013; Cui et al., 2016). CP has also been reported to increase levels of acid phosphatase, alkaline phosphatase, oxaloacetic glutamic acid and glutamic pyruvic transaminase in the liver, indicating a toxic effect of this drug on this vital organ (Cengiz et al., 2019). Following CP administration, the enzyme lipoprotein lipase dysfunction and hepatotoxicity would result in hyperlipidemia and ROS generation. An increase in oxidizing agents can change the profile of fat and cholesterol by changing the oxidation of cholesterol (Senthilkumar et al., 2006). In the present study, administration of CP increased serum levels of total cholesterol, triglycerides, and LDL and decreased HDL. On the other hand, one of the beneficial effects of *M. sativa* is its anti-hyperlipidemia effect. Previous studies have shown that saponins in *M. sativa* reduce cholesterol absorption, and atherosclerotic plaque formation in animals, and *M. sativa* administration reduces plasma and liver cholesterol levels (Bora and Sharma, 2011). The high content of omega-3 fatty acids, phytosterols, and the combination of stigma sterol (as an inhibitor of cholesterol absorption) may improve hyperlipidemia (Seida et al., 2015). In the present study, *M. sativa* administration in MS 200, and 400 groups improved CP-induced hyperlipidemia, and hepatotoxicity.

In the present study, *M. sativa* administration at a dose of 400 mg/kg prevented CP-induced oxidative stress and increased antioxidant defense factors such as total thiol groups, and catalase, and SOD activities in both serum and liver tissue, which is in line with the previously reported evidence for the protective effect of antioxidants against the toxicity of CP (Tripathi and Jena, 2010; Zarei and Shivanandappa, 2013). The presence of vitamin E, and phenolic and flavonoid compounds in *M. sativa* has been related to its functional properties such as antioxidant, anti-inflammatory, and inhibition of xanthine oxidase (Karimi et al., 2013).

Corticosteroids are commonly used for the treatment of thrombocytopenia and as adjutant drugs with CP (Zunjar et al., 2016; Ramadan et al., 2012). However, administration of corticosteroids leads to numerous side effects, including suppression of the immune system (Cines and Blanchette, 2002). Therefore, the use of herbal products as an adjutant to chemotherapy drugs has been considered in recent studies, because they often have anti-inflammatory, anti-tumor, and antioxidant effects and reduce the toxicity of drugs (Cui et al., 2016; Kamali et al., 2018). In the current study, the therapeutic effects of *M. sativa* at the dose of 400 mg/kg were comparable to dexamethasone.

Moreover, the results of the present study showed that *M. sativa* extract does not cause dexamethasone side effects such as impaired lipid profile and elevation of some liver enzymes, and the levels of nitric oxide metabolites.

Previous studies showed no toxic effects for *M. sativa* even at higher dose (Al-Snafi et al., 2021). As limitations, the histopathological evaluation of liver, kidney, and bone marrow, and measurement of kidney biomarkers had been not done in this study, and it is recommended to be considered in future studies.

 The extract of *M. sativa* (especially at a dose of 400 mg/kg) showed therapeutic effects against the CP-induced myelosuppression, thrombocytopenia and an increase in oxidative stress markers which was comparable to the effect of dexamethasone.

## Conflicts of interest

The authors have declared that there is no conflict of interest.
